# Discrepancy Between Invasive and Echocardiographic Transvalvular Gradients After TAVI Procedure: A Review of the Literature

**DOI:** 10.3390/jcm15072740

**Published:** 2026-04-04

**Authors:** Dimitrios Afendoulis, Panayotis K. Vlachakis, Nikolaos Tsiamis, Flora Tsakirian, Christos Tountas, Panagiotis Theofilis, Andreas Synetos, Sotirios Tsalamandris, Fotios Toulgaridis, Maria Drakopoulou, Konstantina Aggeli, Konstantinos Tsioufis, Konstantinos Toutouzas

**Affiliations:** 1 Unit of Structural and Valvular Heart Diseases, First Department of Cardiology, National and Kapodestrian University of Athens, General Hospital of Athens “Hippokration”, 115 27 Athens, Greece; dimitrisafendoulis@yahoo.com (D.A.); nik.tsiamis@gmail.com (N.T.); loratsakirianmed@gmail.com (F.T.); panos.theofilis@hotmail.com (P.T.); stsalamandris@hotmail.com (S.T.); mdrakopoulou@hotmail.com (M.D.); kaggeli@gmail.com (K.A.); ktsioufis@gmail.com (K.T.); ktoutouz@gmail.com (K.T.); 2 Catheterization Laboratory, General Hospital of Athens “Sismanogleio”, 151 26 Athens, Greece; tountasxristos@yahoo.gr (C.T.); fotistoulgaridis@gmail.com (F.T.); 3 School of Medicine, European University of Cyprus, 2404 Nicosia, Cyprus; synetos@yahoo.com

**Keywords:** TAVI procedure, invasive gradient, echocardiographic gradient, transvalvular gradient

## Abstract

**Background/Objectives:** Transcatheter aortic valve implantation (TAVI) has become an established treatment for patients with severe aortic stenosis. The accurate post-procedural assessment of transvalvular gradients is essential for evaluating procedural success and long-term prognosis. However, significant discrepancies have been reported between gradients measured invasively and those derived by Doppler echocardiography. This systematic review aims to summarize the current evidence comparing invasive and echocardiographic gradient measurements after TAVI. **Methods:** A comprehensive literature search was conducted of the PubMed database from inception to 8 November 2025 using the keywords: “TAVI/TAVR,” “invasive versus echocardiographic gradient,” and related terms. Studies were included if they compared invasive and Doppler-derived aortic valve gradients following TAVI. Out of 44 identified articles, 12 studies met the inclusion criteria and were analyzed. **Results:** Across all the included studies, the echocardiography-derived mean gradients were consistently 4–7 mmHg higher than those obtained invasively, reflecting physiologic rather than procedural discordance. The difference was more pronounced in balloon-expandable and small-diameter valves and in patients with high-flow states. Invasive gradients were independently associated with mortality and major adverse cardiovascular events (MACEs) in multiple studies. An invasive mean gradient ≤ 10 mmHg immediately post-TAVI was repeatedly identified as the threshold for optimal procedural success and improved long-term outcomes. **Conclusions:** Doppler echocardiography systematically overestimates transvalvular gradients after TAVI. While both modalities remain valuable, an invasive hemodynamic assessment provides the most reliable evaluation of immediate procedural success and long-term prognosis. Echocardiographic gradients should be interpreted relative to the baseline invasive measurement to avoid overdiagnosis of prosthetic dysfunction and ensure appropriate clinical management.

## 1. Introduction

Transcatheter aortic valve implantation (TAVI) has become the standard treatment for symptomatic severe aortic stenosis in high-risk and elderly patients, with transvalvular gradient measurements serving as critical indicators of procedural success and hemodynamic valve performance [1]. Post-procedural gradient assessment guides clinical decision-making, including the need for post-dilatation or optimization techniques, and provides prognostic information for patient outcomes [1].

The measurement discordance between invasive and echocardiography-derived gradients represents a fundamental clinical challenge in post-TAVI assessment. Echocardiography, which remains the main tool in current clinical practice, consistently overestimates gradients compared to invasive measurements both before and after TAVI, a phenomenon explained by pressure recovery, Bernoulli equation limitations, and differences in hemodynamic conditions during measurement acquisition [1,2]. These technical factors create a clinically relevant discordance that may influence therapeutic decisions and prognostic assessments when the two modalities yield divergent results.

The aim of our review was to summarize the data in the literature regarding which modality provides superior prognostic information and to identify the factors that amplify measurement discordance, which would substantially improve risk stratification and guide appropriate clinical management in the post-TAVI population [1,3,4,5].

## 2. Measurement Methods and Technical Discordance

Invasive gradient measurements rely on the simultaneous recording of left ventricular and ascending aortic pressures using pigtail catheters positioned in the middle of the left ventricle and ascending aorta, respectively [6]. The mean gradients are calculated by integrating the differences in blood pressure between the ventricle and aorta during systole, providing a direct real-time hemodynamic assessment immediately following valve implantation [6]. This invasive approach captures the true transvalvular pressure without accounting for pressure recovery, representing the genuine hemodynamic load imposed on the left ventricle and serving as the gold standard for hemodynamic assessment [2].

Echocardiographic gradient derivation employs continuous-wave Doppler velocities subjected to the simplified Bernoulli equation, which fundamentally assumes a non-laminar flat velocity profile while neglecting the pressure recovery, flow acceleration phenomena, and left ventricular outflow tract contributions [7]. These mathematical and physiological assumptions systematically lead to gradient overestimation, particularly in normally functioning prostheses. Additionally, echocardiographic measurements are obtained under different hemodynamic and positional conditions than invasive assessments: patients are positioned left lateral recumbent during echocardiography, whereas invasive measurements are acquired supine [8]. The timing differences between modalities further complicate their direct comparison, as echocardiographic studies may be performed several days post-intervention in mobilized patients, whereas invasive measurements are conducted immediately post-TAVI during catheterization.

## 3. Materials and Methods

The current review is reported in accordance with the preferred reporting items for systematic reviews (PRISMA). A comprehensive literature search was conducted of the PubMed and Scopus databases from inception to 8 November 2025 to identify the relevant studies. The search strategy included the following keywords: ‘Transcatheter Aortic Valve Implantation, ‘TAVR’, ‘TAVI’, ‘Transthoracic versus transcatheter aortic valve gradient’, and ‘Invasive versus echocardiographic aortic valve gradient’. The Boolean research terms included [“TAVI”/”TAVR”] AND [‘Transcatheter versus transthoracic aortic valve gradient’], [‘Invasive versus echocardiographic aortic valve gradient’]. Synonyms and equivalent terms for these keywords were also included, and the reference lists of the articles included in the review were screened for additional citations to ensure a broad research.

Studies were eligible for inclusion if they focused on TAVR, TAVI, and the evaluation of invasive versus echocardiographic-measured gradient. This included the influence of the TAVI procedure on the echocardiographic-measured gradient postoperatively compared with the transcatheter-acquired gradient during the procedure, and the discordance between the two measurements. The exclusion criteria included abstracts, editorials, case reports, animal studies, and non-English articles. Moreover, any articles that did not consider the relevance of the effect of TAVI procedure on the invasive and echocardiographic aortic valve gradients were excluded from our review. After removing duplicate articles, all available articles were screened for title and abstract relevance. Furthermore, full texts of potentially eligible studies were assessed by the same reviewers. The key data were extracted by the reviewers, focusing on the study and population characteristics.

All titles and abstracts were screened independently by two reviewers of our team (T.F., N.T.), and data extraction was performed. Any conflicts were reviewed by the inclusion of one separate expert reviewer (S.T.) and resolved by consensus of the whole team. The methodological quality was assessed by two more reviewers. Any disagreements regarding classifications were resolved through consensus among the reviewers.

## 4. Results

**Figure 1:**
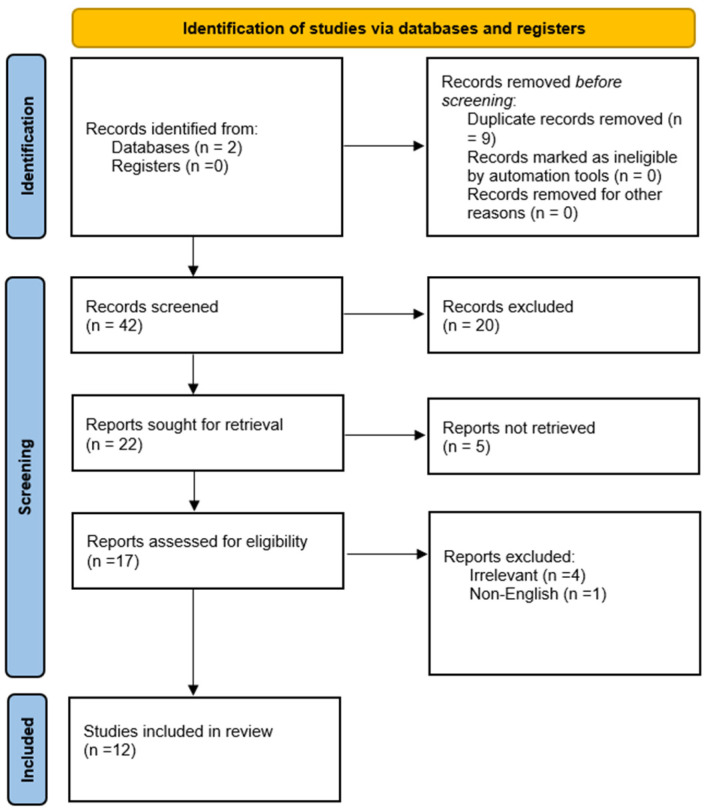
PRISMA flowchart.

According to the results of our review, the data in the literature regarding evaluation of invasive aortic valve gradients versus echocardiographic-measured gradients after TAVI procedures remain limited. Out of the 51 articles we found during our detailed research, after removing duplicate articles, 42 were screened. Finally, 12 articles met our inclusion criteria and were included in our review. The PRISMA flowchart is provided below (Figure 1) (studies summarized in Table 1).

### 4.1. Agreement and Discordance Between Invasive and Echocardiographic Gradients After TAVI

Several studies have consistently demonstrated a systematic discrepancy between invasive and Doppler-derived mean transvalvular gradients following TAVI, with echocardiography tending to overestimate the residual obstruction. In a prospective study by Abbas et al. (2019) [2], the invasive and echocardiographic gradients were measured immediately post-procedure in 160 patients. The authors observed that the Doppler-derived gradients were, on average, 5 mmHg higher than the invasive values, with wide limits of agreement and only a moderate correlation between the two techniques. This difference persisted across valve types and deployment depths, suggesting a physiological rather than procedural cause [2].

Expanding on these findings, Abbas et al. (2021) [7] analyzed 250 patients who underwent both intraprocedural Doppler and invasive assessments. Again, the echocardiographic gradients exceeded the invasive measurements by approximately 4–6 mmHg, and the discrepancy was most evident in balloon-expandable valves and in similar prosthesis sizes. Factors such as higher-flow states and smaller valve diameters independently predicted larger differences, reinforcing that Doppler gradients are inherently flow-dependent and susceptible to overestimation under high-output conditions [7].

Similarly, El- Hachem et al. (2025) [8] examined over 300 patients treated with various modern TAVI prostheses and confirmed a consistent 4–7 mmHg higher mean gradient by Doppler compared with invasive catheterization. The degree of discordance varied according to prosthesis type and size, being greatest in balloon-expandable and small-diameter valves. Importantly, this pattern was not associated with adverse outcomes, indicating a benign, device-related physiologic phenomenon [8]. Additional real-world registry data from the LAPLACE-TAVI Registry confirm that the echo–invasive gradient difference of 4–7 mmHg persists across valve types and sizes, reinforcing the device/size-dependence of the phenomenon [9]. A large cohort study of 507 TAVI patients demonstrated that Doppler-derived mean gradients were significantly higher than catheterization values (e.g., 11.0 ± 5.8 mmHg vs. 3.2 ± 4.0 mmHg in balloon-expandable devices), with the largest discrepancies seen in smaller prostheses and balloon-expandable platforms [10].

Finally, Abbas et al. (2023) [11] broadened the scope by comparing echo and invasive gradients across multiple clinical settings, including native and prosthetic valves. Within the TAVI subgroup, the same ~5 mmHg overestimation of Doppler gradients was observed, accentuated in high-flow states and smaller prostheses. The authors emphasized that such discrepancies were systematic and physiologic rather than device specific, highlighting the need for cautious interpretation of elevated echo gradients when the invasive values are within normal limits [11].

Summarizing, these studies consistently demonstrate that Doppler echocardiography overestimates post-TAVI gradients by 4–7 mmHg on average, with greater discordance in small, balloon-expandable valves and in patients with high flow. The phenomenon reflects inherent physiological factors—pressure recovery, flow acceleration and differences in measurement timing—rather than prosthetic dysfunction, underscoring the importance of using an invasive assessment as the reference standard for immediate post-procedural valve evaluation.

### 4.2. Prognostic Implications of Invasive and Echocardiographic Gradients After TAVI

Beyond the question of agreement, several studies have assessed which modality—echocardiography or invasive catheterization—better predicts clinical outcomes after TAVI. In a large multicenter investigation, Van den Dorpel et al. (2025) [1] demonstrated that although the Doppler gradients were consistently higher than the invasive measurements, only the invasive mean gradient independently predicted long-term mortality and major adverse cardiovascular events (MACEs). After adjustment for valve type, left ventricular function, and stroke volume, the echo-derived gradient lost its prognostic significance, emphasizing that invasive hemodynamics more accurately reflect the true prosthetic performance and patient prognosis [1].

Similarly, Pfenniger et al. (2024) [6] prospectively analyzed over 200 patients who underwent paired invasive and echocardiographic assessments before and after TAVI. While the pre-TAVI correlations between the modalities were strong, the relationship weakened substantially post-implantation, with echocardiography overestimating the gradients by roughly 5 mmHg. Crucially, only the invasive post-procedural gradient was associated with subsequent mortality and heart failure readmission, whereas the echo gradient showed no independent predictive value. The authors concluded that invasive assessment should anchor post-TAVI hemodynamic evaluation and guide early clinical decision-making [6].

The long-term data further supports the prognostic superiority of invasive measurements. In a five-year follow-up study, Murray et al. (2020) [12] observed that the baseline invasive gradients averaged approximately 8 mmHg, while the early echocardiographic gradients were 4–6 mmHg higher. Despite this discrepancy, the Doppler gradients remained stable over time and did not predict valve degeneration or mortality. Instead, patients with initially favorable invasive gradients maintained excellent valve function beyond five years, confirming the durability of results derived from accurate invasive hemodynamic assessment [12].

**Figure 2:**
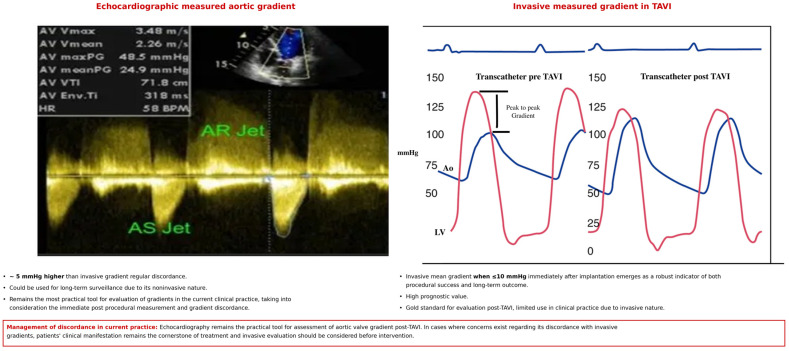
Central illustration. Echocardiographic vs. invasive gradient measurements and suggested treatment algorithm.

Collectively, these studies indicate that while echocardiographic gradients provide convenient non-invasive follow-up data, their prognostic value is inferior to that of invasive measurements. The invasive mean gradient—particularly when ≤10 mmHg immediately after implantation—emerges as a robust indicator of both procedural success and long-term outcomes, whereas the isolated elevation of echo gradients often lacks clinical consequences when the invasive parameters are within a normal range (Figure 2—Central Illustration).

## 5. Discussion

Pre-TAVI gradients demonstrate a strong correlation between invasive and echocardiographic measurements (r = 0.69–0.70), yet post-TAVI the correlation deteriorates markedly to weak levels (r = 0.18–0.23) [6,11]. This dramatic collapse in correlation indicates a functional discordance between modalities after valve implantation, rendering them non-interchangeable for post-procedural assessment. The absolute mean differences between invasive and echocardiographic gradients are quantitatively smaller pre-TAVI (approximately 3–5 mmHg), but remain surprisingly consistent post-TAVI (approximately 5 mmHg), which is reported as a statistically significant result in most studies [6]. However, the proportional percentage discordance increases substantially post-TAVI because the invasive gradients decline more steeply than the echocardiographic measurements, creating clinically meaningful divergence when the absolute differences are expressed as percentage errors.

The transition from a strong to weak correlation post-TAVI reflects fundamental differences in how each modality responds to the hemodynamic environment created by a normally functioning prosthesis. At baseline, when native stenotic valves generate substantial turbulent flow and high velocities across the valves, both methods capture a similar relative severity. Post-implantation, the prosthesis normalizes flow patterns and reduces absolute velocities, but the Bernoulli equation’s sensitivity to velocity changes (which relate non-linearly through the V^2^ term) diverges from direct pressure measurements. As part of the Bernoulli equation, flow acceleration leads to underestimation of echo-measured gradient, while pressure recovery becomes a dominant factor post-TAVI because the reduced turbulence and improved geometric efficiency of the prosthesis allow for greater restoration of kinetic energy to the static pressure downstream of the valve, a phenomenon captured by invasive measurements but systematically overestimated by echocardiography. These technical discordances establish that invasive and echocardiographic gradients measure fundamentally different hemodynamic constructs post-TAVI and necessitate separate prognostic evaluation frameworks.

### 5.1. Valve Type-Specific Discordance and Hemodynamic Differences

The invasive mean gradients immediately post-TAVI are remarkably similar between self-expanding valves (SEVs) and balloon-expandable valves (BEVs), with the median values approximating 3.0 mmHg for both platforms [1,3]. This equivalence in invasive hemodynamic performance between valve designs indicates that direct pressure measurement does not discriminate acute valve function based on the valve architecture. The consistency of invasive gradients across valve types has been confirmed in multiple multicenter prospective studies and propensity-matched analyses [7], establishing that invasive hemodynamics provide a platform-neutral assessment of immediate procedural success and do not preferentially favor either BEV or SEV technology based on the transvalvular pressure differences.

Echocardiography-derived gradients, by contrast, demonstrate substantial differences between valve types post-TAVI that do not reflect true hemodynamic disparities, but rather the differential effects of valve geometry on the Doppler velocity patterns. Balloon-expandable valves exhibit higher mean echocardiographic gradients (11.0 mmHg) compared to self-expanding valves (8.0 mmHg), a 3 mmHg differential that disappears when the same measurements are obtained invasively [1]. Small balloon-expandable valves show particularly pronounced echocardiographic gradient elevation compared to large BEVs, whereas small and large self-expanding valves demonstrate comparable echocardiographic gradients, indicating that valve size-dependent geometric factors drive the discordance [8]. The proportionally smaller orifice area and more restrictive hemodynamic envelope of smaller BEVs generate higher velocity jets that are mathematically amplified by the Bernoulli equation, creating echocardiographic artifacts unrelated to true prosthetic dysfunction.

The magnitude of discordance between modalities is significantly amplified for balloon-expandable valves compared to self-expanding valves, with the median discordance reaching 8.0 mmHg for BEVs versus 5.0 mmHg for SEVs [7]. This differential discordance is driven by a smaller valve size, higher ejection fraction, and higher stroke volume, which amplify the non-linear Doppler velocity effects and consequently enlarge the gap between invasive and echocardiographic measurements [1]. The valve-specific discordance pattern has critical clinical implications for post-discharge echocardiographic interpretation and management decisions. Because elevated discharge echocardiographic gradients in BEV patients may largely represent methodological overestimation rather than true hemodynamic compromise, documented baseline discordance allows clinicians to establish expected gradient ranges and distinguish genuine prosthetic dysfunction from expected measurement variation [1,7]. When follow-up echocardiography is performed on SEV patients, a lower anticipated baseline discordance provides greater confidence that an echocardiographic gradient elevation represents true hemodynamic progression rather than an artifact [7].

### 5.2. Hazard Ratios for All-Cause Mortality by Gradient Type and Analysis Method

A spline curve analysis revealed an inflection point at an invasive gradient of >10 mmHg, identifying this threshold as the clinically meaningful cutoff for increased mortality risk and substantiating its use in clinical decision-making [1]. However, the relationship between invasive gradients and outcomes is non-linear, with Pfenniger and colleagues demonstrating that very low invasive gradients (<3 mmHg) and very high invasive gradients (>6 mmHg) are both associated with excess mortality and major adverse cardiac events [6]. This U-shaped relationship suggests an optimal invasive gradient range of 5–10 mmHg for the best clinical outcomes, indicating that extremely low gradients may reflect hemodynamic compromise or other complications, while high gradients represent true valve-related hemodynamic burden, which was proved with a statistical significance in most studies.

### 5.3. Key Clinical Implications of Gradient–Outcome Associations

- Optimal Invasive Gradient Range: Target an invasive mean gradient between 5 and 10 mmHg post-TAVI; invasive gradients >10 mmHg require consideration of repeat post-dilatation or other optimization strategies to reduce mortality risk [1]. Very low gradients (<3 mmHg) warrant investigation for potential hemodynamic complications or patient–prosthesis mismatch.
- Mortality Risk Stratification: Invasive gradients enable immediate risk stratification, with gradients >10 mmHg identifying patients at significantly increased 30-day, 1-year, and 2-year mortality compared to gradients ≤10 mmHg [1]. This prognostic capability should be leveraged in post-TAVI decision-making, including intensity of follow-up monitoring and optimization of comorbidity management.
- Echocardiographic Discordance Recognition: Discharge or immediate post-TAVI/intraprocedural echocardiographic gradients substantially exceeding invasive measurements should not trigger urgent intervention or heightened clinical concern if the invasive hemodynamics are acceptable [7]. The systematic overestimation by echocardiography makes baseline discharge gradients unreliable for prognostication and potentially misleading for clinical management.
- Procedural Optimization Guidance: Invasive gradient measurement performed immediately post-TAVI in the catheterization laboratory enables real-time optimization decisions, allowing for the correction of inadequate deployment before case completion [7]. Deferring gradient assessment until the discharge echocardiography delays identification of hemodynamically significant malpositioning and misses the opportunity for acute procedural correction.
- Follow-up Echocardiographic Interpretation: Subsequent echocardiographic follow-up should interpret gradient changes in the context of baseline post-TAVI invasive measurements rather than the discharge echocardiographic values; an absolute echocardiographic gradient increase of >3–4 mmHg may represent clinically significant progression when the invasive measurements increase by >1–2 mmHg [6]. This approach prevents overdiagnosis of valve deterioration due to measurement variability or technical factors.
- Platform-Independent Prognostic Value: The prognostic significance of invasive gradients is consistent for both self-expanding and balloon-expandable valves, allowing for uniform risk stratification across TAVI platforms and enabling comparison of patient outcomes across different valve types [1]. Echocardiographic platform-dependent discordance limits its utility for cross-platform outcome comparison.

### 5.4. Structural Valve Deterioration, Patient–Prosthesis Mismatch, and Long-Term Outcomes

Invasive and echocardiographic gradients show limited prognostic utility for predicting bioprosthetic valve failure and structural valve deterioration at the 1-year follow-up, with invasive gradients demonstrating no significant association with these durability complications [1]. A longer follow-up period of at least 6–7 years is typically needed to accurately assess the connection between invasive and echo gradients post-TAVI as predictors of bioprosthetic valve failure, given the fact that these valves typically remain fully functional for a minimum of five years. Most studies in the literature do not demonstrate such a long follow-up of patients, which remains a limitation for our review. Echocardiographic gradients similarly fail to identify patients who will develop valve dysfunction, indicating that a discharge gradient assessment by either modality has insufficient discriminatory power for identifying which patients require enhanced surveillance or early reintervention for hemodynamic deterioration [6]. This finding carries important clinical implications: elevated discharge gradients, regardless of modality, should not independently drive clinical decision-making regarding durability concerns, as neither measurement predicts short-term structural complications.

Patient–prosthesis mismatch (PPM), defined by an indexed aortic valve area below 0.85 cm^2^/m^2^, shows no significant association with 5-year mortality or major adverse cardiac events despite substantial proportions of patients experiencing moderate-to-severe PPM in the perioperative period [6]. The absence of prognostic significance persists through long-term follow-up, suggesting that hemodynamic compromise from PPM does not translate into measurable clinical harm over a 5-year horizon [6]. Notably, the echocardiographic assessment of PPM appears to systematically overestimate the clinical significance, further supporting reliance on invasive hemodynamic measurements when risk stratification is essential for guiding patient management and follow-up intensity.

Long-term echocardiographic follow-up demonstrates remarkable hemodynamic stability in transcatheter-implanted valves, with the mean aortic gradients remaining essentially unchanged from discharge through 5-year follow-up at approximately 9–11 mmHg, reflecting minimal hemodynamic progression over time [12]. Despite this stability, echocardiographic measurements consistently remain approximately 5 mmHg higher than the immediate post-TAVI invasive measurements would predict, confirming that the baseline discordance persists throughout follow-up and that echocardiographic gradient elevations should be contextually interpreted in light of known systematic overestimation. Despite this, in cases of extreme measured gradients of more than 20–30 mmHg, given the insufficient data for the immediate postoperative period, patients should be treated according to their clinical status and not solely based on the gradient discordance. Clinically significant structural valve deterioration occurs at very low rates, with only 8.9% moderate and 1.3% severe deterioration evident at 7 years or longer follow-up, and bioprosthetic valve failure affects just 3.8% of patients. These durability findings provide substantial reassurance regarding long-term transcatheter valve performance and establish that gradient discordance observed at discharge does not indicate compromised prosthesis longevity or a predisposure to accelerated structural failure.

Recent data from large multicenter registries highlight a complex and non-linear relationship between transvalvular pressure gradients and mortality following TAVR, underscoring the significant discordance between echocardiographic and invasive measurements. According to the study by Khalili et al., a low discharge echocardiographic mean gradient (<10 mm Hg) is paradoxically associated with an increased 2-year mortality, a finding primarily driven by its association with low-flow states and reduced left ventricular ejection fraction (LVEF). In contrast, a low invasive gradient (<5 mm Hg) was found to be a marker of improved survival. Similarly, in the context of TAVI-in-SAVR, Kherallah et al. reported that the clinical outcomes follow a U-shaped pattern, with higher event rates at the extremes of post-procedural gradients (<10 and ≥30 mm Hg) and the best prognoses observed within the 10–30 mm Hg range. Furthermore, the data from the registry by Eng et al. confirm that while small (20 mm) balloon-expandable valves (BEVs) are associated with higher discharge echocardiographic gradients compared to larger valves (≥23 mm), they exhibit identical 3-year survival. This suggests that echocardiography may overestimate functional stenosis due to factors such as pressure recovery and flow dependence. Collectively, these findings indicate that the clinical assessment of an isolated elevated echocardiographic gradient post-TAVR is insufficient without accounting for the flow and LVEF, making invasive hemodynamic confirmation essential before considering reintervention [4,5,13,14,15].

To summarize these data, according to the results of our review, it is indicated that while echocardiographic gradients provide convenient non-invasive follow-up data, their prognostic value is inferior to that of invasive measurements. The invasive mean gradient—particularly when ≤10 mmHg immediately after implantation—emerges as a robust indicator of both procedural success and long-term outcomes, whereas the isolated elevation of echo gradients often lacks clinical consequence when the invasive parameters are within a normal range [3].

### 5.5. Future Perspectives: The Role of Artificial Intelligence (AI)

Future advancements in transcatheter aortic valve implantation (TAVI) assessment are likely to be driven by artificial intelligence (AI) and machine learning technologies. AI-based models have the potential to integrate multimodal data—including invasive hemodynamics, Doppler echocardiographic parameters, computed tomography-derived anatomical metrics, and clinical outcomes—to create predictive tools capable of identifying patients at risk of suboptimal valve performance or post-procedural complications. Recent evidence shows that AI models can outperform traditional risk scores at predicting outcomes after TAVI (e.g., pooled AUC ~0.79 vs. ~0.61) [16]. Deep-learning algorithms trained on large-scale imaging and hemodynamic datasets could enable automated detection of discordance between invasive and echocardiography-derived gradients, reducing diagnostic uncertainty and guiding personalized post-TAVI follow-up strategies. On the imaging front, AI-driven segmentation, landmark detection and morphology assessment of the aortic root and prosthesis behavior have shown very high accuracy (e.g., Dice scores >0.90; measurement discrepancies within ±2 mm) in pre-procedural planning [17]. Furthermore, AI-driven simulation of flow dynamics and device–tissue interaction may assist in valve selection and deployment optimization, minimizing the need for invasive reassessment and improving long-term prosthetic durability. The integration of such computational intelligence into clinical workflows promises to refine procedural precision, enhance patient-specific risk stratification, and ultimately redefine the standard for post-TAVI evaluation and management.

## 6. Conclusions

In conclusion, our review summarizes the existing data regarding measurements of invasive gradient versus echocardiographic gradient in patients after a transcatheter aortic valve implantation. The immediate post valve implantation gradient measured in the catheter laboratory remains the gold standard for the evaluation of procedural success and long-term events, and its fall to below 10 mmHg is crucial for a successful operation. The echocardiographic-measured gradient after this procedure demonstrates discordance with the invasive values and is elevated approximately 5 mmHg compared with the invasive gradient due to the changes in flow and hemodynamics caused by the prosthetic valve. Knowing this, the baseline echocardiographic gradient should be measured and used for patient follow-up given its non-procedural nature and high availability, despite its low prognostic value. When evaluating a patient with degeneration of the valve, the patient’s symptoms should be taken into consideration as well, along with the echocardiographic gradient, and an invasive gradient confirmation should be done before any intervention.

## Figures and Tables

**Table 1:** Summary of studies included in our review.

Authors	Year	Study Design	Sample Size	Key Finding	Subject
Abbas et al. [2]	2019	Prospective study	160	Doppler-derived gradients were ~5 mmHg higher than invasive values.	Post-TAVI gradient comparison
Abbas et al. [7]	2021	Prospective analysis	250	Echocardiographic gradients exceeded invasive measurements by 4–6 mmHg; most evident in balloon-expandable valves.	Post-TAVI gradient discrepancy by valve type
El-Hachem et al. [8]	2025	Observational study	300	Consistent 4–7 mmHg higher mean gradient by Doppler vs. invasive; greatest in balloon-expandable and small-diameter valves.	Post-TAVI gradient assessment across prosthesis types
LAPLACE-TAVI Registry [9]	2023	Registry study	2888	Echo–invasive gradient difference of 4–7 mmHg persists across valve types and sizes.	Real-world registry validation of gradient discordance
Biersmith et al. [10]	2022	Cohort study	507	Doppler-derived mean gradients significantly higher than catheterization values (11.0 ± 5.8 vs. 3.2 ± 4.0 mmHg).	TAVI gradient comparison in balloon-expandable devices
Abbas et al. [11]	2023	Cross-sectional analysis	5027	~5 mmHg overestimation of Doppler gradients; accentuated in high-flow states and smaller prostheses.	Echo vs. invasive gradients across clinical settings
Van den Dorpel et al. [1]	2025	Large multicenter investigation	872	Only invasive mean gradient independently predicted long-term mortality and MACEs; echo gradient lost prognostic significance.	Prognostic value of invasive vs. echocardiographic gradients
Pfenniger et al. [6]	2024	Prospective analysis	200	Only invasive post-procedural gradient was associated with mortality and heart failure readmission.	Pre- and post-TAVI gradient comparison with clinical outcomes
Murray et al. [12]	2020	Five-year follow-up study cohort	452	Baseline invasive gradients ~8 mmHg vs. echocardiographic 4–6 mmHg higher; invasive gradients predicted long-term outcomes.	Long-term valve durability and gradient stability
Khalili et al. [13]	2022	Registry study	2251	Low discharge echocardiographic gradient (<10 mmHg) associated with increased 2-year mortality in low-flow states.	Transvalvular pressure gradients and mortality following TAVR
Kherallah et al. [14]	2024	Registry study	12,122	U-shaped relationship with event rates at extremes (<10 and ≥30 mmHg); best prognosis at 10–30 mmHg.	Elevated gradients after TAVI for degenerated surgical aortic valve
Eng et al. [15]	2024	Outcome comparison study	16,200	Small (20 mm) BEVs show higher discharge gradients but identical 3-year survival vs. larger valves.	3-year outcomes of balloon-expandable valves by size

## Data Availability

All data generated or analyzed during this study are included in this published article. Data supporting our review can be found across the two research platforms used for our review (Pubmed, GoogleScholar) and across all the articles of the references section.
